# Occupational Stress in Spanish Police Officers: Validating the Effort-Reward Imbalance Questionnaire

**DOI:** 10.3390/ijerph18041393

**Published:** 2021-02-03

**Authors:** Lourdes Luceño-Moreno, Beatriz Talavera-Velasco, Marian Jaén-Díaz, Jesús Martín-García

**Affiliations:** 1 Department of Social and Work Psychology and Individual Differences, Faculty of Psychology, Complutense University of Madrid, 28223 Madrid, Spain; jemartin@ucm.es; 2 Department of Education, Faculty of Languages and Education, Universidad Antonio de Nebrija, 28015 Madrid, Spain; btalavera@nebrija.es; 3 Department of Business Management, Faculty of Economics and Business Sciences, Pontificia Comillas University, 28015 Madrid, Spain; mjaen@comillas.edu

**Keywords:** job stress, police, occupational health, harm reduction, assessment, ERIQ

## Abstract

The Effort-Reward Imbalance Questionnaire (hereinafter, ERIQ) has been largely used worldwide to assess job stress, but it has not yet been applied in Spanish police. The objective of this study was to examine the construct validity and the internal consistency of the ERIQ in police officers. A cross-sectional study was carried out, using a nonprobability sampling (quota). A total of 217 Spanish police officers participated, 192 men (88.47%) and 25 women (11.53%). The mean age was 41 years (*SD* = 7.51). These police officers completed the ERIQ together with some other questionnaires (DECORE-21, MBI, GHQ and STAI) in order to provide evidence for validity based on the relationships to other constructs. A confirmatory factor analysis was performed and a matrix of correlations with the rest of constructs was created. The results showed an appropriate fit to the original model consisting of three scales. In addition, the scales of the ERIQ presented the expected relationship with the other constructs. The ERIQ is a valid instrument for assessing occupational stress in Spanish police officers and can improve the interventions in this professional group.

## 1. Introduction

The Effort-Reward Imbalance Questionnaire (ERIQ) is based on the theoretical model of the same name, the Effort-Reward Imbalance model [1], which investigates job stress through the imbalance between the efforts the worker makes (costs) and the rewards he or she receives (gains). The effort dimension refers both to: “extrinsic effort” or the demands from the work itself, and “intrinsic effort”, which refers to the motivation of workers in relation to such demands. The rewards that employees receive for their efforts come from three sources: money, esteem, and job security [2]. Intrinsic effort refers to individual differences in the perception of effort-reward imbalance (ERI). Employees with a motivational pattern characterized by high job commitment and high need for approval (over-committed subjects) have an increased risk of experiencing stress (strain) as a result of a non-symmetrical exchange. This seems to be due to these individuals exposing themselves more frequently to high demands at work or exaggerating their efforts; as a result, they develop high expectations for rewards, and are therefore more vulnerable to frustration. These workers are also characterized by exaggerated motivation and unusual high expectations regarding job demands, which leads them to assume greater responsibilities and commit to tighter deadlines.

The main assumptions of the Effort-Reward Imbalance model are the following [3]: (1) Each of the components: effort, reward, and over-commitment (OC), separately, contribute to reducing the health and well-being of workers. The imbalance between high efforts and low rewards (ERI: non-reciprocity situation) produces adverse health effects, which will be added to those already caused by each of the components alone. The most adverse consequences occur when there is a mismatch between the efforts the worker makes and the rewards he or she receives (structural component of the model); (2) an over-commitment acts as an intrinsic trigger for the perception of imbalance between the efforts invested and the rewards obtained in return (person-specific component of the model); and, finally, (3) the most adverse effects on health and well-being will arise when both the structural component and the person-specific component are present.

Some conditions, both from the work context (structural) or the employee’s characteristics (person-specific), may alter these predictions. For example, job instability (structural) or feelings of helplessness (person-specific) may increase adverse effects, while a stable and satisfactory social network (structural) or a high level of self-efficacy and independence (person-specific) reduce such detrimental effects [3]. Empirical evidence for the Effort-Reward Imbalance model [1,2] comes from the studies investigating the association between job stress (strain) understood as ERI, and some diseases, mainly cardiovascular disease (CVD) and its associated risk factors. Relevant research on this association between the ERI model and CVD refer to the studies with London civil servants. Those studies aimed to understand the causes of the variability in disease development between different jobs (The Whitehall II Study) [4,5]. A large amount of information was obtained, more specifically: quality of health, job stress, social support, health habits, stressful events, and mental health. The researchers concluded workers who suffered stressful events (ERI) were twice as likely to suffer CVD compared to those not exposed to the imbalance [4]. Other studies with a similar sample of workers confirmed the results: employees who scored high in the effort/reward ratio were at approximately twice the risk of myocardial infarction compared to those not experiencing such imbalance [6]. A systematic review and meta-analysis revised 22 studies analyzing the association between the ERI model and CVD markers. The results concluded that the ERI and OC were associated with hypertension, fibrinogen and alterations of the intima media thickness [7]. The link between stress (understood as ERI), and some other types of disease has also been studied, in particular: mental health problems [8], somatic complaints, depression [9], diabetes [10], and poor self-assessment of health [11]. In addition, the perception of imbalance between efforts and rewards has been associated to suicidal thoughts. In a study evaluating 416 health care workers, it was determined that the ERI was directly related to suicidal ideation (odds ratio = 1.91, 95% confidence interval [CI] = 1.48, 2.65) [12].

Policing has been found to be amongst the most stressful occupations; police officers must constantly be on alert, as a mistake may have serious consequences either for themselves or others. Police officers are in continuous contact with traumatic events, which makes these workers prone to experience post-traumatic stress [13,14]. In addition, almost one out of two report a medium or high level of burnout [15]. However, they do not only report a more adverse impact of work on health, but also on leisure opportunities and economic well-being, even when compared to firefighters, another high-risk occupation [16]. Some of the job-related stress factors that are frequently reported by police officers are: high job demands, few resources, low autonomy in decision-making, poor relationship with superiors, and other organizational experiences [17,18,19]. The ERIQ assesses some of the stress risk factors involved in police work [20]. Some studies have shown that the ERI has physiological effects, which can contribute to the development of stress-related diseases in police officers [21,22]. It has also been associated with symptoms of anxiety and depression in these professionals [23]. For example, some authors identified that those officers who perceived an imbalance between effort and reward were more likely to suffer from depression (OR 7.89, 95% CI 2.32–26.82), compared with their counterparts who did not perceive the imbalance [17]. Likewise, in a recent study, both the ERI and OC were positively associated with burnout in police officers [24]. The ERIQ has been validated in many countries. In Spain, specifically, it has been validated using a sample of 298 workers from a public hospital in Asturias [25]. However, it is necessary to administer the questionnaire in our country to samples of professionals other than the original one. In addition, it is recommended to examine the psychometric properties of the assessment tool with different samples over time [26]. A review of the use of burnout and stress questionnaires in police [27] concluded that of 111 studies, only 4 had been carried out in Spain: two of them used the Maslach Burnout Inventory (MBI) [15], one used the Social Work Stress Appreciation Scale [28], and another study used the Demands-Control-Rewards (DECORE-21) questionnaire [29].

The relevance of this study lies in the fact that, to date, the ERIQ had not been validated in Spanish police officers. The main reasons to carry out the present study can be summarized as follows. In the first place, several organizations, such as the American Education Research Association (AERA), the American Psychological Association (APA), and the National Council on Measurement in Education (NCME) recommend to examine the psychometric properties of the assessment instruments in different samples over time [26]. Secondly, no previous studies assessing ERI in Spanish police officers were found, except for the questionnaires used in our study.

Given the negative consequences of stress and the need for valid and reliable assessment tools to measure job stress, the present study aimed to validate the ERIQ in Spanish police officers. Other measures such as psychosocial risks, burnout, self-perceived health and trait anxiety were used to assess the concurrent and discriminant validity of the questionnaire.

The hypotheses are as follows:

**Hypothesis** **1** **(H1).** *The ERIQ will have good psychometric properties in police officers, with a three-factor structure (effort, reward and over-commitment).*

**Hypothesis** **2** **(H2).** *The police officers that perceive high efforts, low rewards or are highly over-committed will have higher levels of anxiety, a worse perception of their health or higher levels of burnout.*

## 2. Materials and Methods

### 2.1. Participants

For this cross-sectional study, a nonprobability design with quota sampling was used. The only inclusion criterion for police officers was a minimum seniority in the current position of one year. A total of 217 police officers participated, 192 men (88.47%) and 25 women (11.53%). The mean age was 41 years (standard deviation [hereinafter *SD*] = 7.51), ranging from 28 to 59 years. The average working hours per week was 37.87 (*SD* = 8.79). Mean seniority in their current position was 9.5 years (*SD* = 14), and in average, participants had been in the police force Reviewers 1, 3. Specific hypotheses to clarify the information are included for 15 years (*SD* = 8). For level of education, 48.3% (105) of respondents had a high school diploma, 24% (52) held bachelor’s degrees, 19.4% (42) had completed secondary vocational education, and 8.3% (18) had a middle school diploma. According to professional category, 84.3% (183) were patrol officers, 8.3% (18) corporals, 6.9% (15) non-commissioned officers, and 0.5% (1) police commissioner. All participants had a fixed contract. According to work shifts, 37.3% (81) worked the morning shift, 29% (63) the afternoon shift, 24.4% (53) worked night shift, 5.1% (11) afternoon/night rotating shift, 2.8% (6) morning/night rotating shift, 0.5% (1) morning/afternoon/night rotating shift, and 0.9% (2) worked morning/afternoon rotating shift.

### 2.2. Instruments

ERIQ: The Spanish version of the Effort-Reward Imbalance Questionnaire [25] was used; this 23-item questionnaire assesses the employee’s perception of the efforts the individual makes in relation to the rewards he or she receives in return (ERI). The extrinsic component of the model consists of two scales, the Effort scale (six items) and the Reward scale (11 items). The Effort scale refers to demanding aspects of the work environment (fast pace at work, numerous interruptions while performing job, a great deal of responsibility, need to work overtime, job being physically demanding, and job being more and more demanding). The Reward scale assesses different aspects of the rewards the worker receives for doing his or her work. This scale consists of three other subscales: esteem rewards (five items: lack of recognition from superiors; lack of recognition on the part of coworkers; not being supported in difficult situations; being treated unfairly; and not receiving recognition for effort or achievements); financial and status related (four items: little promotion prospect; mismatch between job category and education and experience; lack of adequate promotion chances; inadequate salary considering effort and achievements); and job security (two items: deteriorating working conditions; job insecurity). Participants rated each item using a five-point Likert-type scale, ranging from 1 = *I disagree*, to 5 = *I agree, and I am very distressed*). The score for effort is obtained by adding the scores on the six items of the scale; therefore, the total score for this scale ranges from 6 to 30. The higher the score, the higher the effort experienced by the employee. The score on the Reward scale is the sum of the items on that scale, so the total score for reward ranges from 11 to 55. The higher the score, the lower the rewards the worker receives. The Over-commitment scale (hereinafter, OC) is measured by adding the score on the six items, which are responded in the same way as those of the extrinsic part of the model. Employees who score high on OC tend to exaggerate job demands and score higher on the Effort scale. The assessment of the psychometric properties of the Spanish version of the ERIQ is satisfactory, with reliability values above 0.80 for the scales, except for OC (0.63), although in Spanish police officers it had not yet been applied; thus, these properties will be analyzed.

To assess the concurrent and discriminant validity of the ERIQ, the following instruments were used:

DECORE-21 Questionnaire [29]: assesses, like the ERIQ, the perception of stress-related psychosocial factors. The scales that make up the questionnaire are: Cognitive Demands, Control, Rewards, and Organizational Support. It has shown adequate psychometric properties with internal consistency values for the scales between 0.67 and 0.92.

MBI-HSS-22 Questionnaire [30,31]: measures burnout in workers and consists of three scales: Emotional Exhaustion, Depersonalization and Personal Fulfillment. This tool was chosen because of its proven good psychometric characteristics, with Cronbach’s alpha values (reliability) ranging from 0.70–0.80 for the different scales. The validity of the questionnaire has been determined by confirmatory factor analysis, indicating a good fit for the three-factor solution.

GHQ-28 Questionnaire [32,33]: it is a measure of how people perceive their health. It consists of four scales: Scale A (Somatic Symptoms), Scale B (Anxiety and Insomnia), Scale C (Social Dysfunction) and Scale D (Severe Depression). Its psychometric properties are adequate, with reliability values for all scales above 0.90.

STAI Questionnaire [34,35], that consists of two scales, State Anxiety and Trait Anxiety. Likewise, its validity is adequate and presents reliability values above 0.90.

### 2.3. Procedure

The participants and their superiors were informed about the voluntary and anonymous nature of the investigation. A written informed consent was collected from each participant before completing the questionnaires, that were administered in paper format; completed questionnaires were returned in sealed envelopes to the researchers. After eliminating cases with missing values, the responses of 217 police officers were finally obtained.

### 2.4. Data Analysis

The data were analyzed using the RStudio interface (RStudio, Inc., Boston, MA, USA) and SPSS 24.0 statistical software (IBM SPSS Version 24.0. Armonk, New York, NY, USA). The psychometric properties of the 23 items of the ERIQ, construct validity and reliability were examined. Confirmatory Factorial Analysis of its items was performed, using the CFI (Comparative Fit Index), TLI (Tucker–Lewis Index), RMSEA (Root mean square error of approximation), and SRMR (standardized root mean square residual) to assess the model fit; Cronbach’s alpha index was used to measure internal consistency. The minimum sample size to carry out the Factor Analysis is 100. However, the ideal would be to have five observations per variable, being the most acceptable size a ratio of 10:1 [36].

## 3. Results

### 3.1. Construct Validity

**Figure 1:**
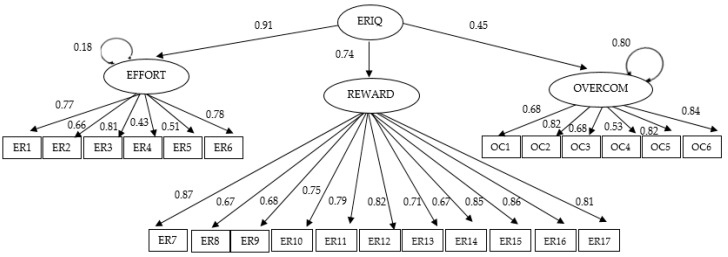
Path diagram of the final three-factor model.

In relation to Construct validity, the CFI, TLI, RMSEA, and SRMR measures were examined in order to assess the fit of the original model of 23 items in this sample of police officers (see Figure 1).

With respect to the fit of the CFI, TLI, RMSEA and SRMR measures, it was confirmed that the 23 original items are grouped into three factors (Effort, Reward and OC). The values of these indices are acceptable (0.98, 0.98, 0.06, and 0.07, respectively). Figure 1 presents the factorial loads for the final model consisting of three factors (see Table 1).

### 3.2. Internal Consistency

Table 2 lists the mean and standard deviation of each item, the correlation between the item and the total scale, and Cronbach’s alpha when the item is deleted. In addition, the mean, standard deviation and Cronbach’s alpha of each of the scales are included. As shown in Table 2, the Cronbach’s alpha indices indicate good levels of internal consistency, ranging from 0.78 to 0.91 for the different subscales of the ERIQ (see Table 2). The internal consistency of a factor is considered acceptable when the Cronbach’s alpha index is 0.70, and it is very good when alpha is 0.80 or greater [37]. In this study, all the dimensions are above 0.80, except for the Effort factor, which has an internal consistency of 0.78 (nearly 0.80).

### 3.3. Evidence of Validity based on the Relationship with other Constructs

Table 3 shows the correlations between all factors in the questionnaires: ERIQ (Effort, Reward and OC), MBI-HSS (Emotional Exhaustion, Depersonalization and Personal Fulfillment), STAI (State Anxiety and Trait Anxiety), DECORE-21 (Cognitive Demands, Control, Rewards, Organizational Support, and Global Risk Score), and GHQ (Somatic Symptoms, Anxiety and Insomnia, Social Dysfunction, and Severe Depression). In addition, the mean and standard deviation of each of them are presented. In relation to the correlations between the scales of the ERIQ, the highest correlation occurs between the Effort and Reward scales, with a value of 0.61 (*p* < 0.01), being the smallest correlation between the Reward and OC scales: 0.30 (*p* < 0.01); the correlation between Effort and OC is 0.31 (*p* < 0.01). With respect to the correlations between the ERIQ Effort scale and the scales of other questionnaires, the following correlations should be highlighted: with Emotional Exhaustion 0.54 (*p <* 0.01) and Depersonalization 0.43 (*p <* 0.01) of the MBI-HSS; with Cognitive Demands 0.41 (*p <* 0.01) and DECORE-21 Global risk score 0.43 (*p <* 0.01); and with Anxiety and Insomnia 0.42 (*p <* 0.01) and total score 0.41 (*p <* 0.01) from the GHQ. The highest correlations between the ERIQ Reward scale and the scales of other questionnaires are: with Emotional Exhaustion 0.55 (*p <* 0.01) of the MBI; with State Anxiety 0.52 (*p <* 0.01) of the STAI; with Organizational Support 0.55 (*p <* 0.01) of the DECORE-21; with Anxiety and Insomnia 0.47 (*p <* 0.01) and total score 0.44 (*p <* 0.01) of the GHQ. Finally, the highest correlations between OC and the scales of the rest of the questionnaires appear to be: with State Anxiety 0.44 (*p <* 0.01) of the STAI, and the Anxiety and Insomnia scale 0.43 (*p <* 0.01) of the GHQ (see Table 3).

## 4. Discussion

This study evaluated the psychometric properties of the ERIQ in Spanish police officers. Results revealed three factors, the same as the original model: effort, reward, and over-commitment. All the model fit indices and the reliability of all the scales were good. The three-factor structure of the ERIQ was also confirmed in epidemiological studies conducted in five European countries, with good fit indices in all cases [38].

There is also an association between the ERIQ factors and other similar constructs such as those evaluated by the DECORE-21 Questionnaire (cognitive demands, control, rewards, and organizational support), and its global risk score. In this sense, the correlation between the ERIQ effort scale and the DECORE-21 Cognitive Demands scale indicates that they both measure similar factors. Likewise, the effort factor is closely related to the emotional exhaustion dimension of MBI-HSS, and this makes sense, since excessive demands have a positive association with exhaustion [39]. The present study has also found a high and significant positive correlation between the ERIQ Effort scale and both the Anxiety and Insomnia scales and the total score of the GHQ. It is remarkable, however, that the correlation between the ERIQ Reward scale and the DECORE-21 Rewards scale, although significant and in the expected direction, is not very high (r 0.29 *p* < 0.01). This may be because the ERIQ Reward scale measures salary, esteem and job security and the DECORE-21 Rewards scale assesses salary and job security, being organizational support (esteem) measured by a different factor. On the other hand, the DECORE Organizational Support scale and the ERIQ Reward scale present a moderately high significant correlation, since the latter assesses organizational support (esteem). With respect to the OC factor it is worth noting that the direct correlation between OC and the GHQ Anxiety and Insomnia scales are high and significant, probably because employees who score high on OC tend to develop more health problems than those who score low. This is also supported by the high and positive relationship found between the ERIQ OC scale and the STAI State Anxiety scale. Previous studies have reported the link between OC and reduced well-being and other pathologies such as musculoskeletal pain or CVD [40]. The ERIQ Reward scale also presents a high and positive relationship with the GHQ Anxiety and Insomnia scale and total risk score, and with the STAI State Anxiety scale. These results show the relationship between poor perception of rewards and health problems. With respect to rewards, similar results were found in a recent experimental study, suggesting that the increase of monetary rewards can reduce stress and, therefore, reduce the risk of ill health, as well as improve job performance [41]. Another aspect to be considered is that the present study also brings out the relationship between emotional exhaustion and the total score of the GHQ. Previous studies with police officers revealed that burnout is a response to the exposure to prolonged stress and can be a serious health threat [42,43]. For example, the results of a recent study with Portuguese police officers revealed that 85% of them suffered from high operational stress levels, 11% had critical burnout values, and 28% had high distress levels, with 55% of the sample at risk of psychological disorders [27]. Our results point to the appropriateness of applying the ERIQ for the assessment of job stress in the Spanish police force. This questionnaire has been widely used in other countries to measure stress in different types of occupational populations, such as firefighters, police officers, specialty physicians, nurses or teaching staff. There is even a Persian and a Thai version of the ERIQ, which were applied to industrial personnel and showed appropriate psychometric characteristics in both cases [44,45]. One of the strengths of this instrument is that, despite not being developed as a clinical evaluation tool, it has revealed an association between its factors and different health conditions (CDV, musculoskeletal, health habits) [46]; thus, its use in the workplace becomes essential. In this regard, other studies have shown that the ERIQ can be used as a predictor of sick absence, considering both the short-term (up to nine days) and long-term (10 days or more) [47]. Regarding the hypotheses described in the introduction, the ERIQ has good construct validity and reliability properties for the sample of Spanish police officers assessed in this study. This means, on the one hand, that hypothesis 1 is correct. On the other hand, hypothesis 2 is correct as well, given the fact that the ERIQ factors are related in the expected direction to other constructs, such as burnout, perceived health and state-trait anxiety. The ERIQ dimensions are also associated, again in the expected direction, with the scales of the DECORE questionnaire. However, the correlation is not very high with some of them because they assess different aspects. One of the limitations of this study is its cross-sectional nature. Aside from this aspect, it would have been more appropriate to obtain a more balanced sample of male and female officers, although this is complicated as women are still under-represented in the police force. With less than 30 participants in the female group, it was not possible to carry out separate statistical analyses for men and women had. In such cases, it is recommended not to conduct separate statistical analyses [48]. Therefore, it is more adequate not to generalize the results of this study to the female police population and more studies in which female officers are better represented should be conducted. In addition to the need for further investigation on the Effort-Reward Imbalance model within the police, future studies should expand the analysis of psychometric properties of the ERIQ in other occupational populations; gaining knowledge about its impact on workers’ health, can help design more effective health promotion interventions.

## 5. Conclusions

This study confirms that the ERIQ is a suitable instrument for assessing occupational stress in Spanish police officers. The analysis of the psychometric properties of the ERIQ shows satisfactory results and the use of the questionnaire among this professional group is therefore recommended. Additionally, the perception of an effort-reward imbalance appears to be associated with burnout, poor health and anxiety. Finally, studies with this population is scarce, which is why this study highlights the importance of assessing occupational stress in police officers for promoting anti-stress interventions.

## Figures and Tables

**Table 1:** Fit Indices obtained in the Confirmatory Factor Analysis using the ERI-Q.

Fit Index	ERI (23 Items)	Accepted Values
CFI	0.987	≥0.90
TLI	0.986	≥0.90
RMSEA	0.066	≤0.05–0.08
SRMR	0.075	≤0.08

Note: CFI = Comparative Fit Index; TLI = Tucker–Lewis Index; RMSEA = root mean square error of approximation; SMR = standardized root mean square residual.

**Table 2:** Descriptive statistics and internal consistency of ERI-Q.

Item	Mean	Standard Deviation	Corrected Item-Total Correlation	Cronbach’s α If Item Deleted	Subscale (α; Mean; *SD*)
ERI_1	2.67	1.04	0.65	0.72	0.78; 14.44; 4.12
ERI_2	2.47	1.04	0.55	0.75	
ERI_3	3.17	1.01	0.68	0.72	
ERI_4	1.89	0.90	0.35	0.79	
ERI_5	1.71	0.89	0.38	0.79	
ERI_6	2.43	1.03	0.62	0.73	
ERI_7	2.65	1.22	0.76	0.90	0.91; 26.44; 9.40
ERI_8	2.16	1.00	0.56	0.91	
ERI_9	2.55	1.27	0.59	0.91	
ERI_10	1.86	1.07	0.64	0.91	
ERI_11	2.65	1.22	0.72	0.91	
ERI_12	2.86	1.17	0.73	0.91	
ERI_13	2.05	1.25	0.59	0.91	
ERI_14	2.07	1.11	0.61	0.91	
ERI_15	2.53	1.13	0.79	0.90	
ERI_16	2.63	1.17	0.79	0.90	
ERI_17	2.44	1.19	0.64	0.91	
OC1	2.07	0.65	0.46	0.80	0.81; 12.09; 3.17
OC2	1.86	0.72	0.68	0.76	
OC3	2.05	0.81	0.53	0.79	
OC4	2.21	0.75	0.45	0.81	
OC5	1.92	0.72	0.71	0.75	
OC6	1.97	0.75	0.65	0.77	

**Table 3:** Mean, standard deviation (*SD*) and correlations between factors.

Factor	1	2	3	4	5	6	7	8	9	10	11	12	13	14	15	16	17	18
1. Effort (E)	-	0.61 **	0.31 **	0.54 **	0.43 **	−0.01	0.37 **	0.24 **	0.35 **	0.16 *	0.33 **	0.41 **	0.43 **	0.39 **	0.42 **	0.26 **	0.16	0.41 **
2. Reward (ERIQ)		-	0.30 **	0.55 **	0.38 **	−0.11	0.52 **	0.31 **	0.55 **	0.30 **	0.38 **	0.28 **	0.54 **	0.37 **	0.47 **	0.27 **	0.24 **	0.44 **
3. OC (ERIQ)			-	0.37 **	0.13	−0.06	0.44 **	0.40 **	0.17 **	0.11	0.28	0.06	0.17 *	0.25 **	0.43 **	0.08	0.19 **	0.33 **
4. Emotional Exh. (MBI)				-	0.51 **	−0.12	0.66 **	0.59 **	0.46 **	0.25 **	0.33 **	0.20 **	0.44 **	0.49 **	0.61 **	0.37 **	0.42 **	0.61 **
5. Depersonaliz. (MBI)					-	−0.23 **	0.34 **	0.29 **	0.33 **	0.60	0.25	0.13	0.31 **	0.23 **	0.31 **	0.15 **	0.19 **	0.29 **
6. Pers. Fulfil. (MBI)						-	−0.36 **	−0.33 **	−0.26 **	−0.01	−0.12	0.13	−0.10	−0.16 **	−0.19 **	−0.21 **	−0.17 **	−0.22 **
7. State anxiety (STAI)							-	0.78 **	0.41 **	0.15 **	0.17 *	0.02	0.27 **	0.52 **	0.67 **	0.50 **	−0.53 **	0.69
8.Trait anxiety (STAI)								-	0.27 **	0.08	−0.09	−0.04	0.14	0.52 **	0.65 **	0.43 **	0.57 **	0.69 **
9. Org. Sup. (DECORE)									-	0.31 **	0.54 **	0.15	0.72 **	0.21 **	0.35 **	0.21 **	0.15 **	0.30 **
10. Rewards (DECORE)										-	0.37 **	0.22 **	0.70 **	0.11 **	0.17 **	0.14 **	0.15 **	0.17 **
11. Control (DECORE)											-	0.25 **	0.80 **	0.14	0.18	0.14	0.11	0.18
12. Cog. D. (DECORE)												-	0.55 **	0.11	0.16	−0.02	−0.13	0.70
13. Global S. (DECORE)													-	0.12 **	0.30 **	0.17 *	0.11	0.26 **
14. Somat. S. (GHQ-A)														-	0.71 **	0.48 **	0.44 **	0.87 **
15. Anx. & Ins. (GHQ-B)															-	0.46 **	0.48 **	0.88 **
16. Soc. dysf. (GHQ-C)																-	0.55 **	0.70 **
17. S. depres. (GHQ-D)																	-	0.72 **
18. Total Score (GHQ)																		-
M	14.43	26.44	12.08	24.76	8.88	35.61	14.51	12.65	247.20	377.62	268.69	325.69	304.81	4.32	4.01	7.10	0.88	16.32
*SD*	4.11	9.45	3.17	10.63	6.15	7.59	9.59	8.91	54.68	64.76	65.75	50.65	41.32	3.53	3.75	1.89	2.32	9.41

Note * *p* < 0.05, ** *p* < 0.01.

## Data Availability

The data presented in this study are available on request from the corresponding author. The data are not publicly available due to the fact that these workers are members of the state security forces. Authors are authorized for academic use, but not for publication, as the data are sensitive.
